# Anxious-depressive symptoms after a first episode of schizophrenia: Response to treatment and psychopathological considerations from a 2-year follow-up study in Italy

**DOI:** 10.1192/j.eurpsy.2024.285

**Published:** 2024-08-27

**Authors:** L. Pelizza, E. Leuci, E. Quattrone, A. Di Lisi

**Affiliations:** ^1^Università di Bologna, Bologna; ^2^AUSL di Parma, Parma; ^3^Alma Mater Studiorum University of Bologna, Bologna, Italy

## Abstract

**Introduction:**

Depression is common in schizophrenia and is correlated with suicide risk and poor long-term outcomes. However, the presence of depressive symptoms is often underestimated in both research and treatment, particularly at the illness onset.

**Objectives:**

The goals of this study were: (a) to longitudinally observe anxious-depressive symptom levels in patients with First Episode Schizophrenia (FES) during a 24 months of follow-up period, and (b) to examine their associations with other psychopathology and the intervention patients received in an “Early Intervention in Psychosis” (EIP) program during the follow-up period.

**Methods:**

The Global Assessment of Functioning (GAF) and the Positive And Negative Syndrome Scale (PANSS) were completed by 159 FES patients both at baseline and across the follow-up. Data were analyzed by linear regression analysis and Spearman’s coefficients.

**Results:**

Anxious-depressive symptoms had significant longitudinal associations with GAF deterioration and PANSS “Positive Symptoms”, “Negative Symptoms” and “Disorganization” subscores. During the follow-up period, FES participants significantly improved the level of anxious-depressive symptoms. This was significantly associated with the number of case management and individual psychotherapy meetings the patient engaged in, as well as with lower antipsychotic doses prescribed during the follow-up period.

**Conclusions:**

In conclusion, anxious-depressive symptoms are prominent in FES and at the initial entry into EIP programs. Anxious-depressive symptom severity tends to diminish overtime, especially with the provision of specialized EIP treatments. However, since we did not have a control population studied in parallel, we cannot say whether these results are specific to the protocols of EIP programs or just to the intensity of engagement in care.

**Disclosure of Interest:**

None Declared

